# 1D CNN-LSTM Based on Quality Classification in Ultrasonic Metal Welding of Multi-Layered Foil Stacks

**DOI:** 10.3390/ma19204357

**Published:** 2026-10-16

**Authors:** Bumsu Go, Kyounghak Kim, Heeseon Bang

**Affiliations:** 1 Research Institute, Korea Welding and Joining Engineering Association, Jeonnam-Gwangju Special Metropolitan City 61012, Republic of Korea; rhqjatn8@naver.com; 2 Department of Welding and Joining Science Engineering, Chosun University, Jeonnam-Gwangju Special Metropolitan City 61452, Republic of Korea; banghs@chosun.ac.kr

**Keywords:** ultrasonic metal welding, Cu foil welding, sensor, classification model, convolutional neural network (CNN), long short-term memory (LSTM)

## Abstract

Ultrasonic metal welding (UMW) is widely utilized for joining multi-layered current collector foil stacks in lithium-ion battery manufacturing, where online joint quality monitoring is critical to prevent defects. In this study, the linear ultrasonic welding of a 60-layer pure Cu foil stack (8 µm thickness each) was investigated by establishing an in situ multi-modal sensing system and a deep learning-based quality classification framework. A linear variable differential transformer (LVDT) sensor and an ultrasonic power meter were synchronized to simultaneously capture mechanical horn displacement and electrical power signals across sequential welding stages. Experimental characterization revealed distinct phenomenological regimes in the acquired profiles, with the vertical displacement showing a direct correlation with the knurl penetration and weld indentation depth. Based on the delivered welding energy, the joint quality was classified into three distinct regimes: under-welding (<500 J), normal welding (500–900 J), and over-welding (>900 J). A two-branch hybrid 1D Convolutional Neural Network (CNN) and Long Short-Term Memory (LSTM) architecture was developed to effectively learn both local transient patterns and long-term temporal dependencies from the preprocessed time-series signals. The proposed classification model achieved an overall prediction accuracy of 98% (macro F1-score of 0.98) on the held-out test set, demonstrating high classification performance for individual welding signals within the investigated process domain. These results demonstrate the feasibility of using multi-sensor in situ data combined with hybrid deep learning models for intelligent, real-time quality assurance in multi-layered Cu foil (8 µm) stack joining.

## 1. Introduction

Driven by the exponential growth of the electric vehicle (EV) and energy storage system (ESS) sectors, requirements for high-capacity and highly reliable lithium-ion batteries (LIBs) have escalated. For pouch-type batteries, welding of multi-layered current collector foil stacks (e.g., commercial pure-grade Cu for anode or Al for cathode) with electrode tabs is a critical manufacturing process that governs overall cell conductivity, structural reliability, and safety.

**Figure 1:**
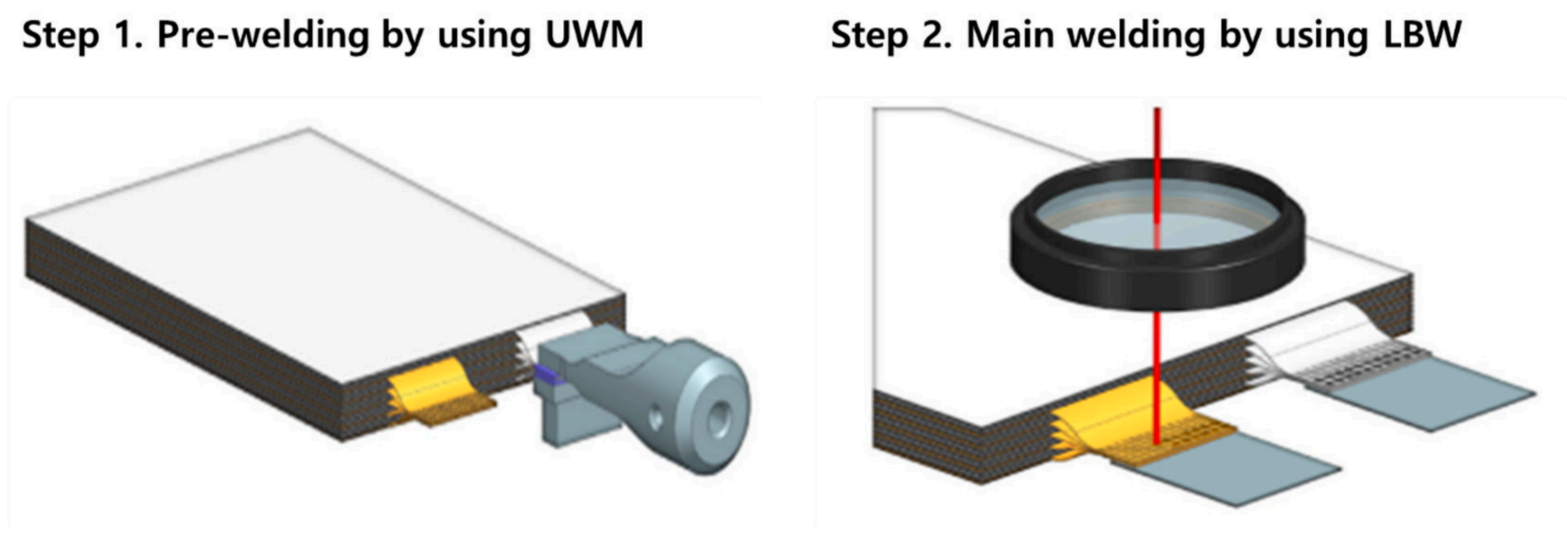
Schematic of pre-welding and main welding was provided by McGovern M. et al. [6].

To ensure the mechanical stability and electrical performance of lithium-ion batteries, various advanced joining techniques have been commonly employed in foil-to-tab interconnections. Among them, ultrasonic metal welding (UMW) is widely utilized due to its ability to handle multi-layered thin foils without inducing excessive thermal damage, relying on high-frequency mechanical vibrations [1,2]. Meanwhile, laser beam welding (LBW) has gained significant attention for its precision, high processing speed, and localized heat input, making it suitable for high-density packaging [3,4]. Additionally, resistance spot welding (RSW) has traditionally been employed for its cost-effectiveness and simplicity, although thermal control remains a key challenge [5]. Therefore, selecting an optimal welding process is a priority for increasing the reliability and performance of battery cells. Because electrode stacks typically comprise multi-layers (consisting of 15 to 50 aluminum and copper foils), their interconnection generally requires a two-step welding approach, in which the foil tabs are initially consolidated via a pre-weld and subsequently joined to the external battery tab lead. This rapid joining process, which is completed within seconds, typically employs individual welding processes or a combination of both (as illustrated schematically in Figure 1). The individual foils are generally on the order of 10 μm in thickness, whereas the tab thickness typically ranges from 0.1 to 0.6 mm [6].

Highlighting the significance of pre-welding in multi-layered copper foil stacks, as shown in Figure 1, several studies have extensively investigated bonding mechanisms, process optimization, thermo-mechanical behavior using finite element modeling (FEM), and deep learning-based quality inspection. Hong et al. [7] performed the optimization of the welding process for 60-layer oxygen-free copper foils (8 μm thick). They identified an optimal welding energy range of 500 to 900 J, demonstrating that exceeding 900 J leads to excessive foil thinning and interfacial failure. Kwon et al. [8] analyzed the thermal and mechanical behavior of the joint region in multi-layer copper foil stacks during ultrasonic welding using a two-dimensional plane strain finite element analysis. They stated that while low clamping pressure and a low vibration amplitude induce stress concentration near the knurl regions, an increase in vibration amplitude promotes a more uniform stress distribution across the joint. This effective dispersion of vibrational energy mitigates localized stress concentrations, thereby enhancing the overall mechanical reliability of the interconnection. Kulkarni et al. [9] investigated bonding mechanisms in ultrasonic welded multi-layered Cu foils (0.1 mm thick Electrolytic Tough Pitch, ETP). They explained that the energy generated during welding significantly contributes to interlayer bonding within the top and bottom regions of the weld nugget through plastic deformation, mechanical interlocking, and material mixing. Furthermore, bonding mechanisms such as micro bonds and interfacial grains were predominantly observed in the middle region of the weld nugget.

Recently, data-driven deep learning approaches, notably 1D convolutional layers and attention mechanisms integrated with LSTM networks, have been extensively applied to predict the weld penetration and to identify weld defects in the monitoring of various welding processes [10,11,12,13,14]. Incorporating a 1D CNN at the input stage of an LSTM framework enables the extraction of robust, noise-filtered features prior to modeling temporal dependencies. Furthermore, 1D convolution operations effectively compress large time-series datasets, reducing the input volume to the LSTM and consequently enhancing overall computational efficiency [15]. Yu et al. [10] proposed a hybrid CNN–LSTM deep learning model to accurately predict weld penetration from dynamic process sequence data during gas tungsten arc welding (GTAW), demonstrating the efficacy of combining CNN local feature extraction with LSTM long-term temporal memory. They reported that the proposed CNN-LSTM model successfully estimates the backside weld bead profile with an accuracy of 0.3 mm using sequential weld pool images from an HDR camera, even under random variations in primary welding parameters. Luo et al. [11] reported a hybrid model combining a 1D convolution layer and LSTM to monitor the penetration state in real time based on acoustic signals in a laser beam welding (LBW) process. This model achieved a prediction accuracy of 99.8% for all three penetration states (insufficient, partial, and full penetration). Chang et al. [12] developed an attention-enhanced 1D CNN-LSTM framework to monitor nugget defects in resistance spot welding (RSW), achieving a high classification accuracy of 98.5%. Furthermore, by visualizing the learned attention weights, they successfully provided physical interpretability for the model’s diagnostic decisions. Ji and Nor [13] proposed a hybrid 1D CNN-LSTM model with an attention mechanism to implement a digital twin system in gas metal arc welding (GMAW) and to monitor the four types of welding defects (insufficient penetration, excessive penetration, undercut, and burn-through) by measuring arc acoustic signals. They showed that the Se-CNN-LSTM model achieved superior results with accuracy of 91% across multiple metrics, including accuracy, recall, precision, area under the curve (AUC), receiver operating characteristic (ROC) and F1 score. Peng et al. [14] proposed an attention mechanism for a model combining a Multi-Scale Convolutional Neural Network (MSCNN) and BiLSTM to monitor the nine types of welding defects (defocus 3 mm, defocus −3 mm, deformation, cracks, repetition, lack of weld, drift, base material tilt, and watermarks). They reported that the MSCNN-BiLSTM-attention model achieved superior results with accuracy of 99.38%. Furthermore, in the context of ultrasonic metal welding for Cu foil stacks and foil-to-tab welding, Hong et al. [16] applied 1D CNNs and Recurrent Neural Networks (RNNs) to time-series process signals to classify the three quality classes (Class 0 for insufficient welding, Class 1 for sufficient welding, and Class 2 for excessive welding) of multi-layer copper foil welds (60 layers for 8 µm thick Cu foil), verifying that learned temporal features outperform traditional manual feature extraction. This model achieved accuracy of approximately 97%. Choi et al. [17] investigated quality prediction algorithms for multi-layered Cu battery joints (40 layers for 8 µm thick Cu foil and 0.2 mm thick Ni-plated Cu strip), establishing a comprehensive evaluation methodology using a support vector machine (SVM)-based algorithm. The accuracy is determined to be approximately 98%.

Although previous studies have explored individual aspects of multi-layer welding, a unified in situ monitoring framework that synchronizes multi-modal physical signals (LVDT displacement and electrical power) and employs a 1D CNN–LSTM architecture for 60-layer ultra-thin Cu foil (8 µm) stacks has not been fully realized. In mass production lines, tool wear and misalignment of the horn or anvil during the pre-welding stage can lead to critical weld defects. Thus, real-time inspection methods are increasingly demanded to ensure joint quality. To address this gap, this study proposes an in situ multi-sensor monitoring system with a two-branch hybrid 1D CNN–LSTM quality classification model.

## 2. Research Methods

### 2.1. In Situ Multi-Modal Sensing and Experimental Setup

**Figure 2:**
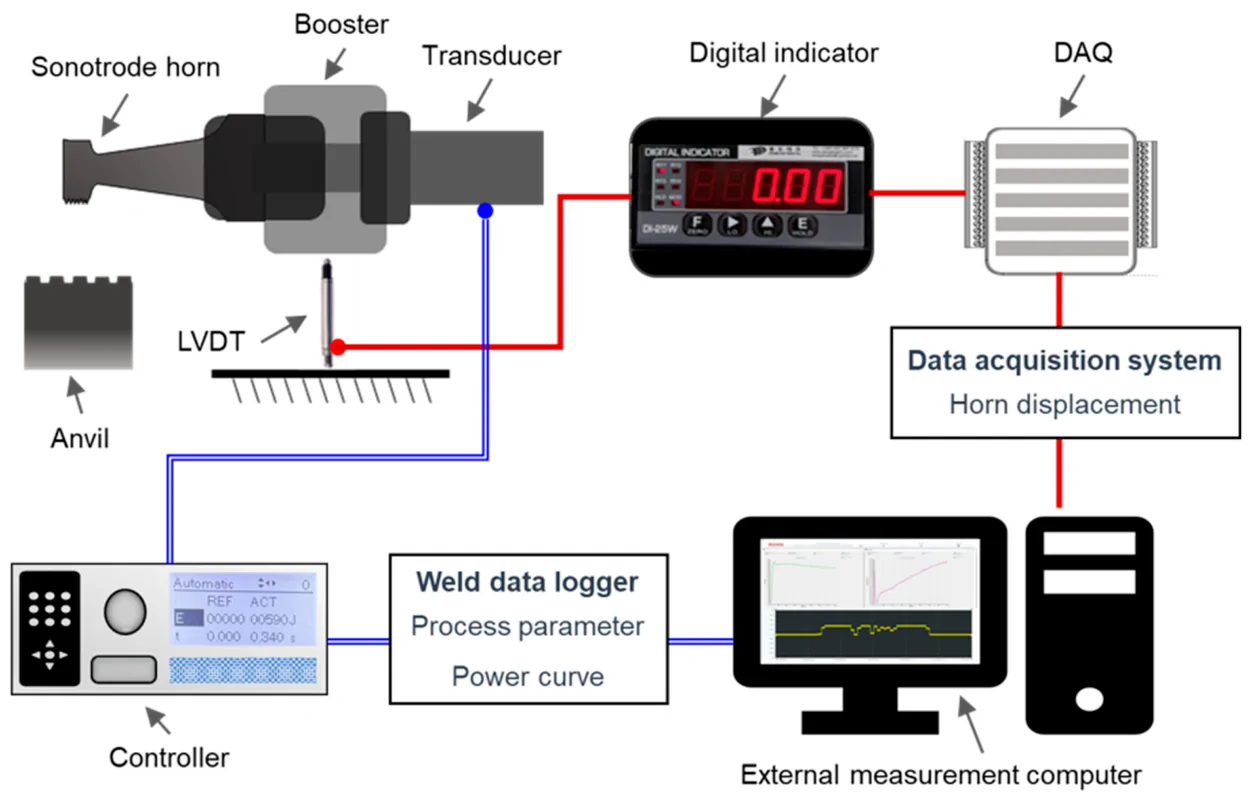
Schematic of experimental setup for process monitoring.

To comprehensively characterize the dynamic physical phenomena governing the ultrasonic metal welding (UMW) process, an in situ multi-modal sensing system was established. The core process parameters—namely, real-time electrical power and vertical tool displacement—were synchronized to capture both the electromechanical response and the mechanical compaction behavior across the joint interfaces. The overall experimental and data acquisition setup is schematically illustrated in Figure 2. A piezoelectric transducer system was utilized to convert high-frequency electrical excitation into linear mechanical vibrations, which were transmitted to the workpiece stack through a booster and sonotrode horn assembly at designated vibrational amplitudes. The instantaneous electrical power delivered to the piezoelectric transducer was measured at the ultrasonic welding controller module and logged using dedicated recording software (Data Recorder G3, version V03.05, Herrmann Ultraschall, Karlsbad, Germany) on an external computer. The technical specifications of the ultrasonic data recorder are summarized in Table 1. Simultaneously, the vertical displacement between the sonotrode horn and the stationary anvil along the clamping axis was monitored using a contact-type linear variable differential transformer (LVDT) probe sensor (DP-10, DONG-DO TECH Co., Ltd., Incheon, Republic of Korea). The LVDT sensor was mounted on the rear side of the sonotrode stack housing within the booster fixture, secured via a magnetic base with an adjustable dovetail clamp. The probe was interfaced with a high-precision digital indicator (DI-20W, DONG-DO TECH Co., Ltd., Republic of Korea). The analog voltage output (0–10 V) from the indicator module was digitized and acquired using a multi-function data acquisition (DAQ) device (NI USB-6001, version 5.1, National Instruments Corp., Austin, TX, USA) operating with DAQ Express software. Data acquisition was strictly synchronized with the welder controller by triggering from the weld release start signal. The detailed technical specifications for the LVDT sensor, digital indicator, and DAQ module are listed in Table 2 and Table 3.

### 2.2. Mathematical Modeling and Physical Principles

During the UMW process, the electrical power delivered by the ultrasonic generator is continuously modulated by the closed-loop controller to sustain a constant vibrational amplitude against time-varying interfacial mechanical loads. The instantaneous mechanical power dissipated at the horn–workpiece interface can be expressed as

*P*(*t*) = *F*(*t*)·(*dS*(*t*)/*dt*) = *μ*(*t*) *F*_N_·(*dS*(*t*)/*dt*) (1)

where *F*(*t*) denotes the instantaneous tangential frictional force exerted by the sonotrode horn, *μ*(*t*) is the dynamic coefficient of friction at the contact interface, *F_N_* represents the applied normal clamping force, and *dS*(*t*)*/dt* is the linear vibrational velocity profile of the sonotrode horn.

Under steady-state process control, the prescribed vibration amplitude is maintained by the closed-loop controller, while the instantaneous vibrational velocity (*dS*(*t*)*/dt*) varies sinusoidally. For a given ultrasonic frequency and amplitude, the peak vibrational velocity remains approximately constant. Therefore, the mechanical power in Equation (1) can be interpreted based on its cycle-averaged behavior. It should be noted that the measured power signal represents the electrical power delivered to the transducer rather than the interfacial mechanical power itself. However, because the electrical power is continuously adjusted to maintain the prescribed vibration amplitude against changes in mechanical loading, the measured power signal can be used as an indirect indicator of changes in the welding process state.

Concurrently, the calibrated LVDT voltage signal was converted into physical displacement using a first-order linear calibration function, V = 0.0338D + 4.975V = 0.0338D + 4.975, where V is the LVDT output voltage and D is the displacement. Accordingly, the displacement was calculated as D = (V − 4.975)/0.0338. The resulting displacement profile provides critical quantitative metrics regarding dynamic plastic deformation, stack compaction, and sonotrode knurl penetration depth, which collectively govern interfacial bonding quality in multi-layered foil stacks.

## 3. Results and Discussion

### 3.1. LVDT Vertical Displacement Profile Analysis

**Figure 3:**
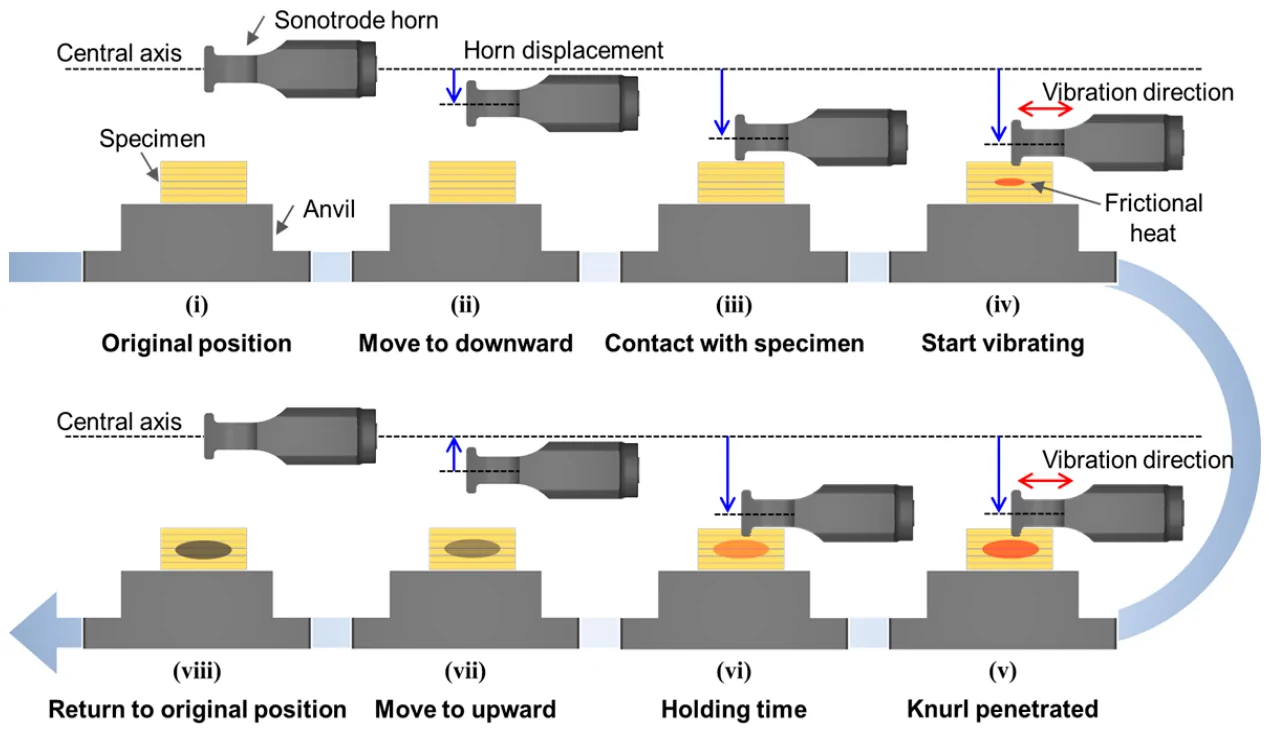
Schematic diagram of LVDT signal.

**Figure 4:**
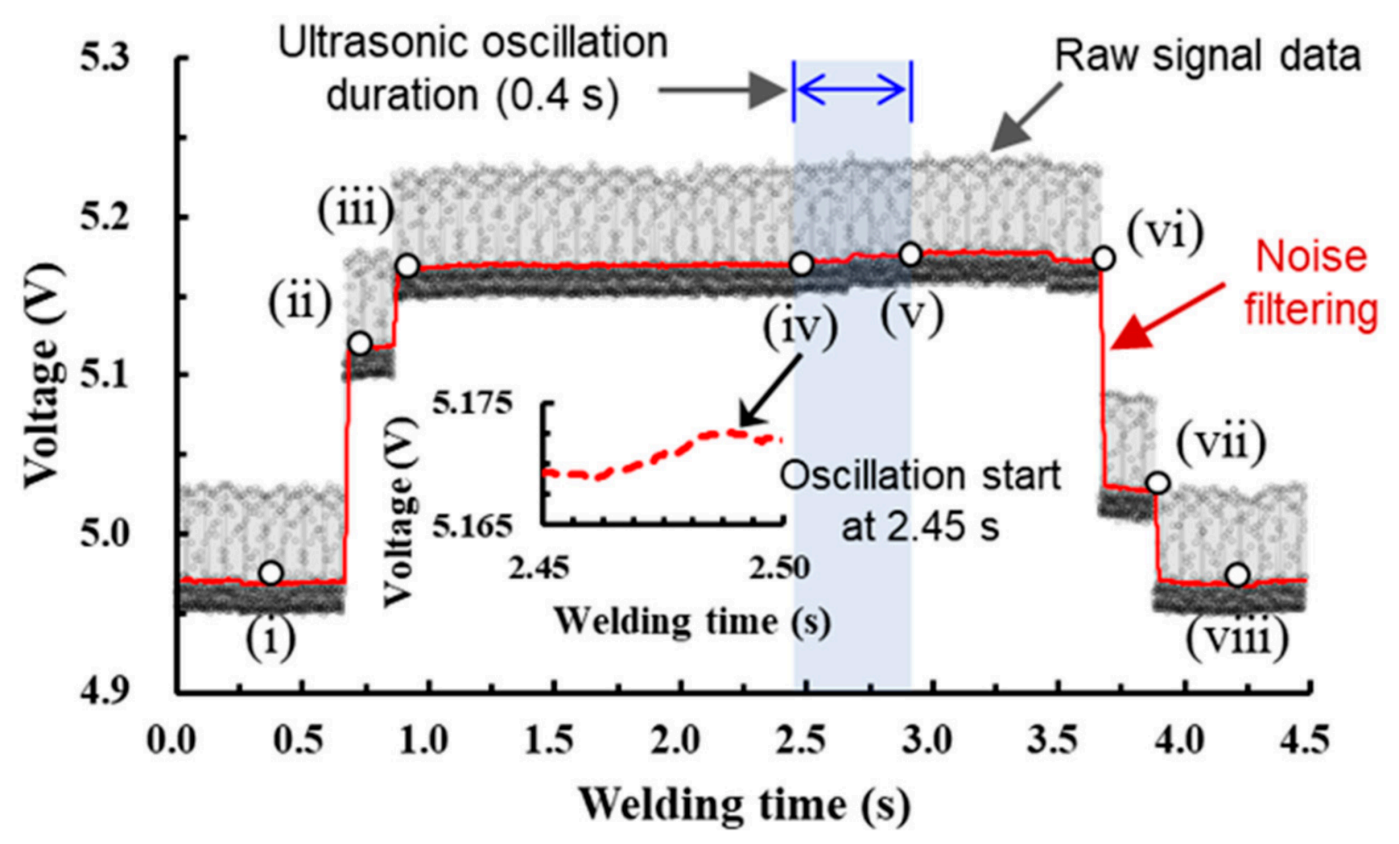
Typical trend of LVDT signal during ultrasonic welding.

**Figure 5:**
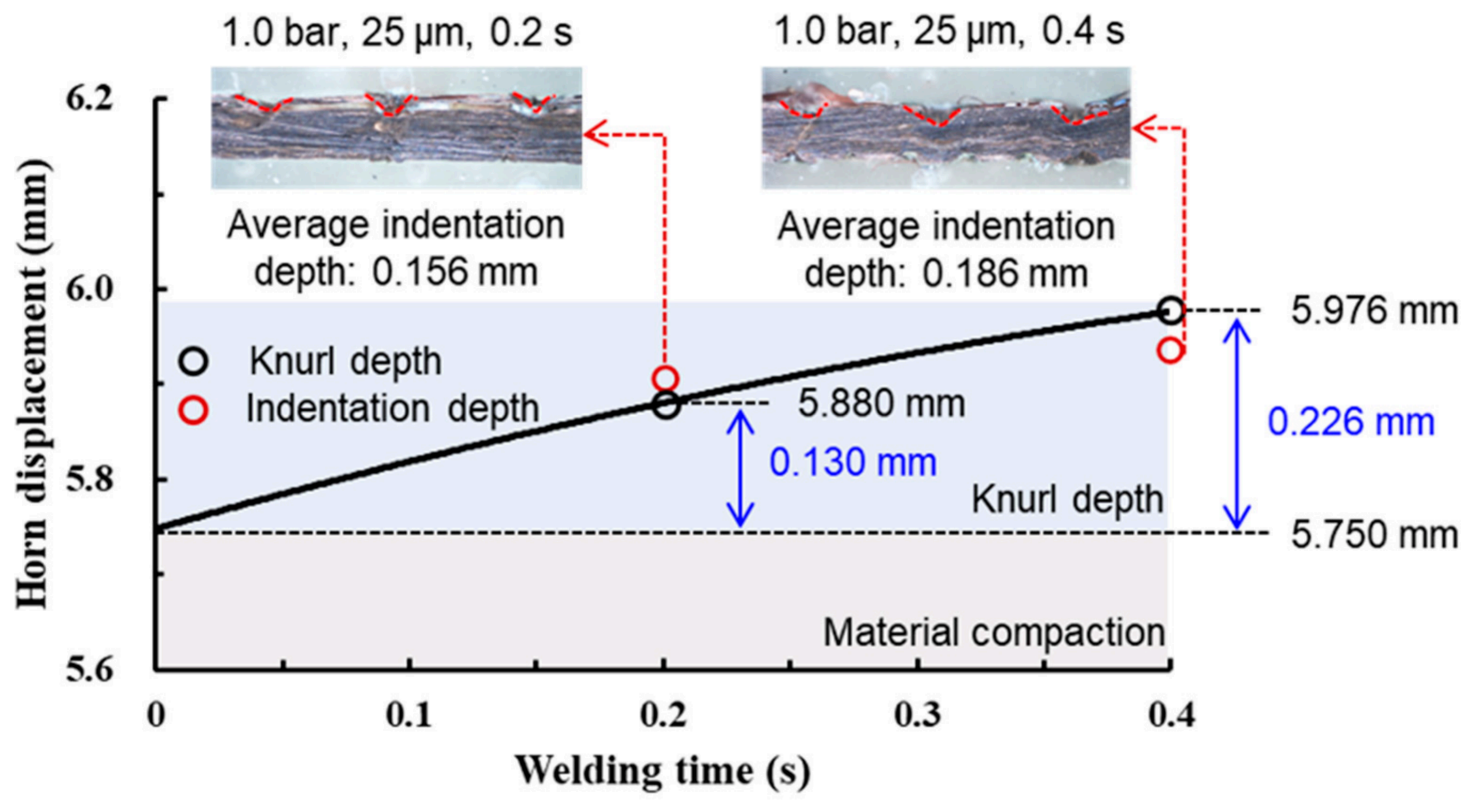
Comparison of horn displacement and indentation depth under varying welding times.

**Figure 6:**
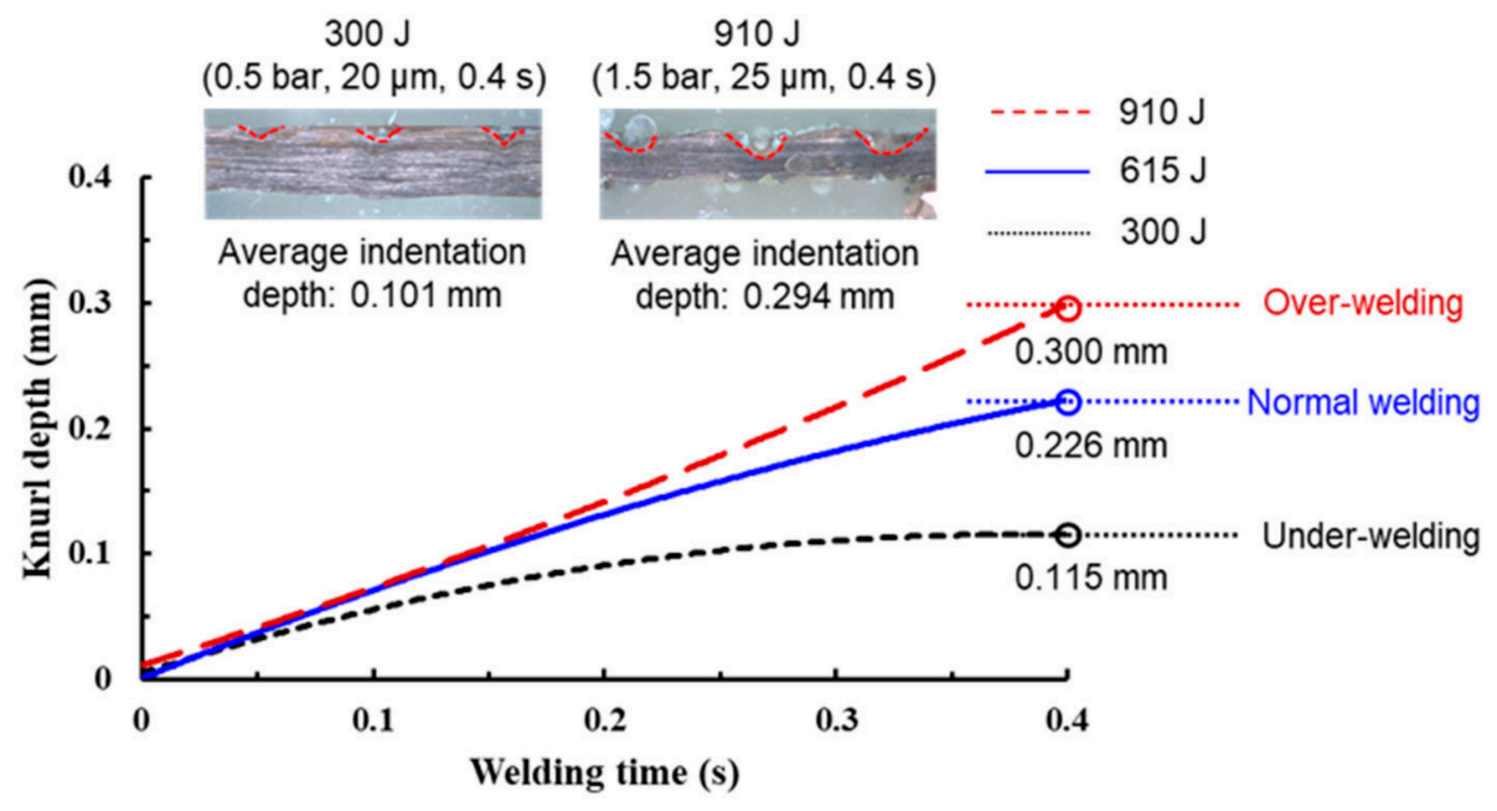
Comparison of LVDT signal with classified welding energies.

The vertical displacement profile obtained from the LVDT sensor directly reflects the sequential mechanical stages of the ultrasonic metal welding (UMW) cycle. As schematically illustrated in Figure 3, the entire joining process is divided into eight distinct stages. A representative LVDT voltage profile recorded under baseline welding conditions (clamping pressure: 1.0 bar, vibrational amplitude: 25 μm, welding time: 0.4 s) is presented in Figure 4. To eliminate high-frequency acoustic interference and acquisition noise, a moving average filter was applied to the raw signal (red curve). During the approach phase (Stages i–ii), the output voltage exhibited a sharp upward step. Upon initial knurl contact (Stage iii), the signal stabilized into a plateau with an approximate slope of zero, followed by a slight gradual increase resulting from the initial elimination of inter-foil gaps. At the onset of ultrasonic oscillation (Stage iv, t = 2.45 s), the slope steepened significantly as vibrational energy induced rapid stack compaction. By the end of oscillation (Stage v), maximum knurl penetration was attained under combined acoustic softening and normal pressure. The displacement remained constant throughout the holding duration (Stage vi) before returning to the baseline upon tool retraction (Stages vii–viii). Quantitative correlation between the measured LVDT displacement and actual cross-sectional weld indentation was established via first-order linear calibration, as depicted in Figure 5. As the welding time increased from 0.2 s to 0.4 s, the measured knurl penetration depth expanded from 0.130 mm to 0.226 mm. Microstructural cross-sectional measurements showed that the corresponding average weld indentation depth increased from 0.156 mm to 0.186 mm. The two measurements exhibited a similar increasing trend, although quantitative differences were observed. This qualitative agreement indicates that the vertical displacement can serve as an in situ physical indicator of weld penetration behavior. Furthermore, Figure 6 compares knurl penetration behaviors across three distinct welding energy regimes:

- Under-welding regime (<500 J): At an energy input of 300 J, the penetration depth was 0.115 mm (average indentation: 0.101 mm), corresponding to less than 30% of the total initial stack thickness. The slope of the knurl penetration depth gradually decreased with increasing welding time.
- Normal welding regime (500–900 J): At an optimal energy of 615 J, the penetration depth grew linearly to 0.226 mm, corresponding to 30–50% of the total initial stack thickness, which yields optimal acoustic energy transmission and metallurgical bonding without tearing.
- Over-welding regime (>900 J): At an excessive energy input of 910 J, the penetration depth reached 0.300 mm (average indentation: 0.294 mm), exceeding 50% of the stack thickness. The penetration rate accelerated nonlinearly toward the end of the cycle, leading to excessive foil thinning, micro-cracking, and mechanical tearing along the knurl periphery.

### 3.2. Ultrasonic Dynamic Electrical Power Signal Characteristics

**Figure 7:**
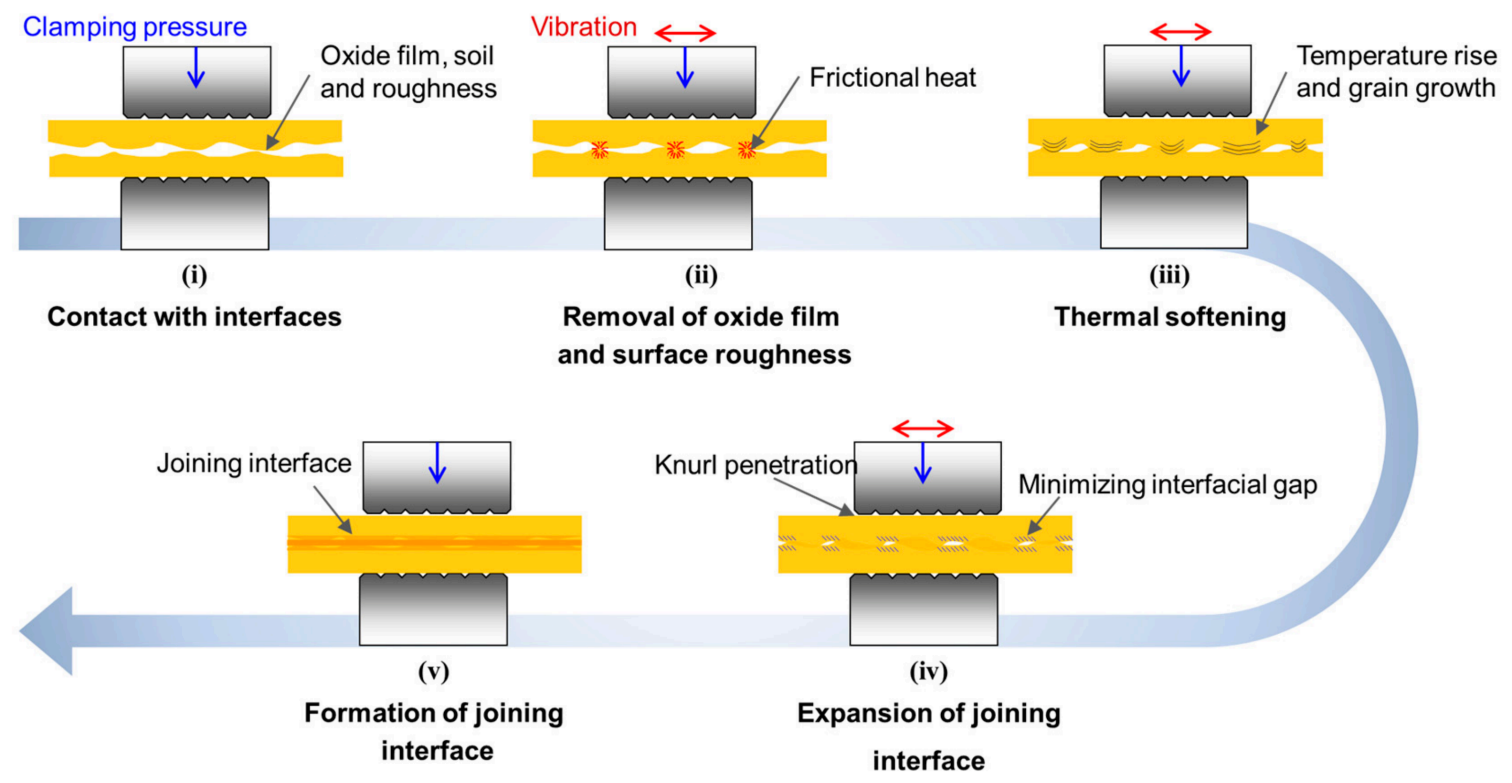
Schematic diagram of power signal.

**Figure 8:**
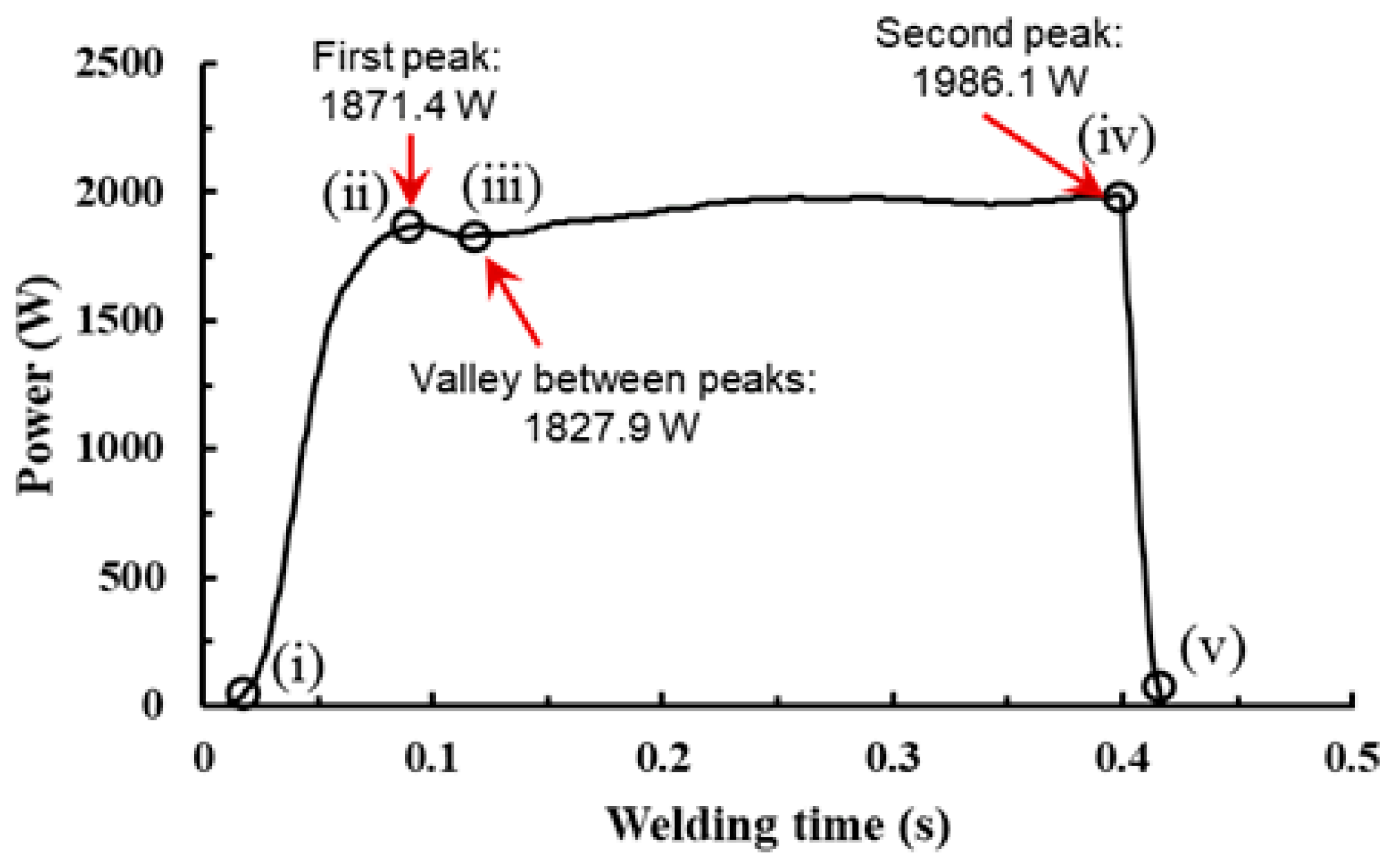
Typical trend of power signal during ultrasonic welding.

**Figure 9:**
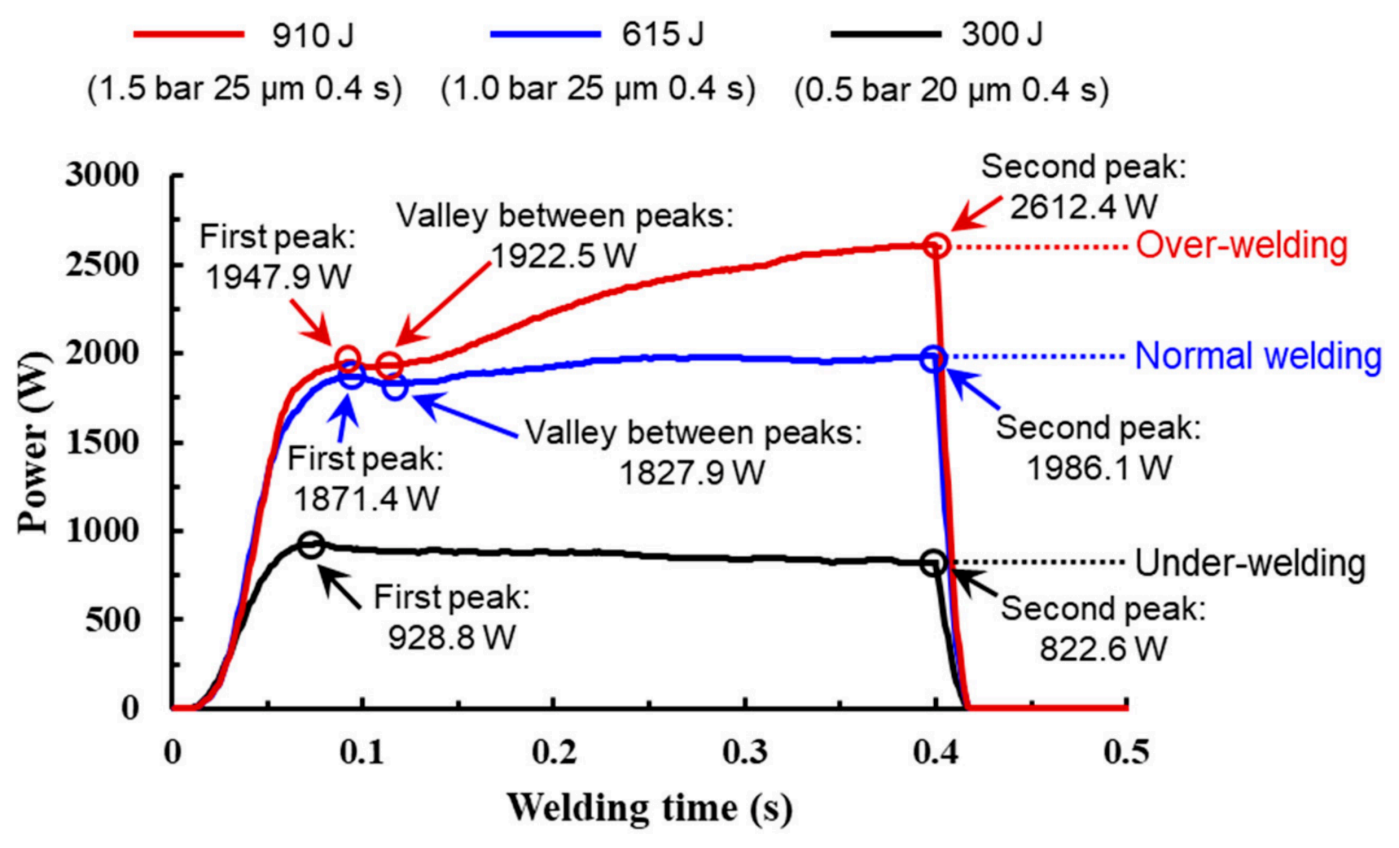
Comparison of power signal with classified welding energies.

The dynamic electrical power profile recorded by the welder controller reflects the transient interfacial friction and thermodynamic state transitions. As illustrated in Figure 7, the power signature is characterized by five consecutive phenomenological stages. Figure 8 presents the characteristic power response for a normal weld (1.0 bar, 25 μm, 0.4 s). The initial peak reached 1871.4 W at t = 0.095 s as interfacial asperities sheared. As thermal softening occurred, the required power declined to a valley of 1827.9 W at t = 0.12 s. Subsequently, as the bonded area expanded under acoustic consolidation, the power rose to a secondary peak of 1986.1 W at t = 0.4 s before plunging upon vibration cessation. The dynamic power behaviors under the three classified energy states are comparatively shown in Figure 9.

- In the under-welding state (300 J), the power peaked at 928.8 W and continuously decayed without exhibiting a distinct secondary rise; the final power level was approximately 11% lower than the initial peak (822.6 W vs. 928.8 W) due to insufficient knurl penetration and limited bonding expansion.
- In the normal welding state (615 J), the signature maintained a balanced two-peak profile with a stable plateau at 1986.1 W.
- In the over-welding state (910 J), the secondary peak surged aggressively without plateauing, reaching 2612.4 W—a 34% increase relative to its initial peak (1947.9 W)—driven by severe plastic deformation and work-hardening around deeply penetrating tool knurls.

### 3.3. Preprocessing of Multi-Sensor Sequential Data

**Figure 10:**
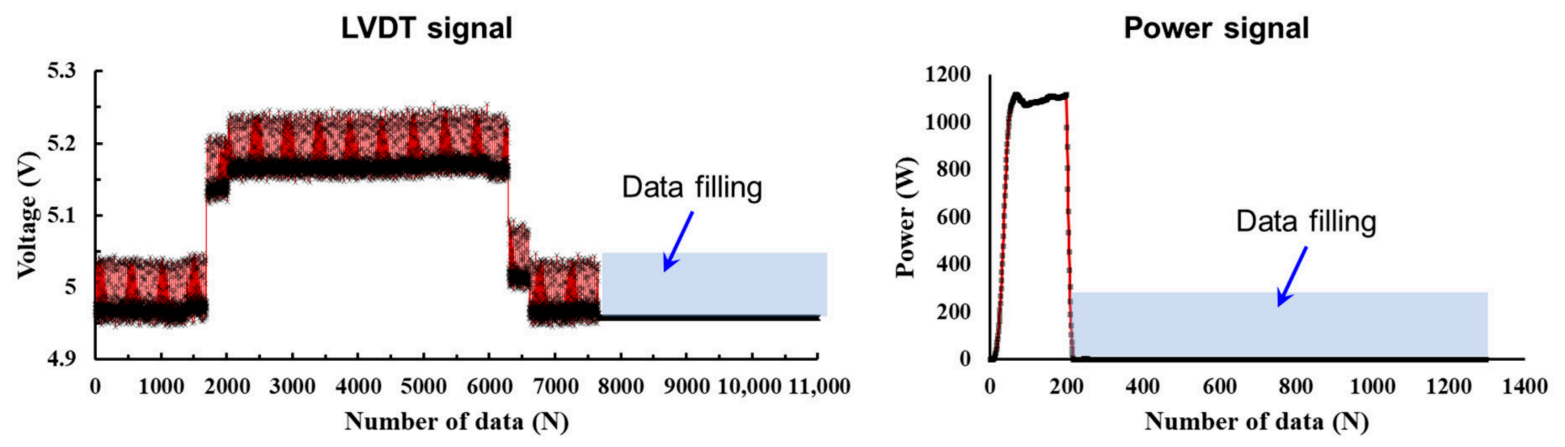
Schematic of preprocessing in LVDT and power signal.

Standardization of input dimensions is required before feeding heterogeneous time-series sequences into deep learning models. Because the welding duration varied from 0.2 s to 0.8 s across different parameter combinations, raw signal lengths differed substantially. To establish a uniform input tensor shape without distorting temporal characteristics, the maximum acquisition duration corresponding to 0.8 s was selected as the baseline reference. As illustrated in Figure 10, the LVDT displacement signal lengths collected at 0.2, 0.4, 0.6, and 0.8 s were 2750, 5500, 8250, and 11,000 data points, respectively. For sequences with welding durations ≤ 0.6 s, the signal was padded to a length of 11,000 points using last-value padding (maintaining steady-state displacement). Similarly, the ultrasonic power profiles containing 699, 899, 1099, and 1299 data points were padded to a standard sequence length of 1299 data points by padding trailing zero-power values.

The experimental database comprises a total of 324 samples generated from 36 distinct process parameter sets with nine repetitions each. The dataset was classified into three supervised quality labels based on delivered welding energy, as detailed in Table 4 and Table 5: Class 0 (Under-welding, <500 J), Class 1 (Normal welding, 500–900 J), and Class 2 (Over-welding, >900 J).

### 3.4. 1D CNN–LSTM Architecture and Training Setup

**Figure 11:**
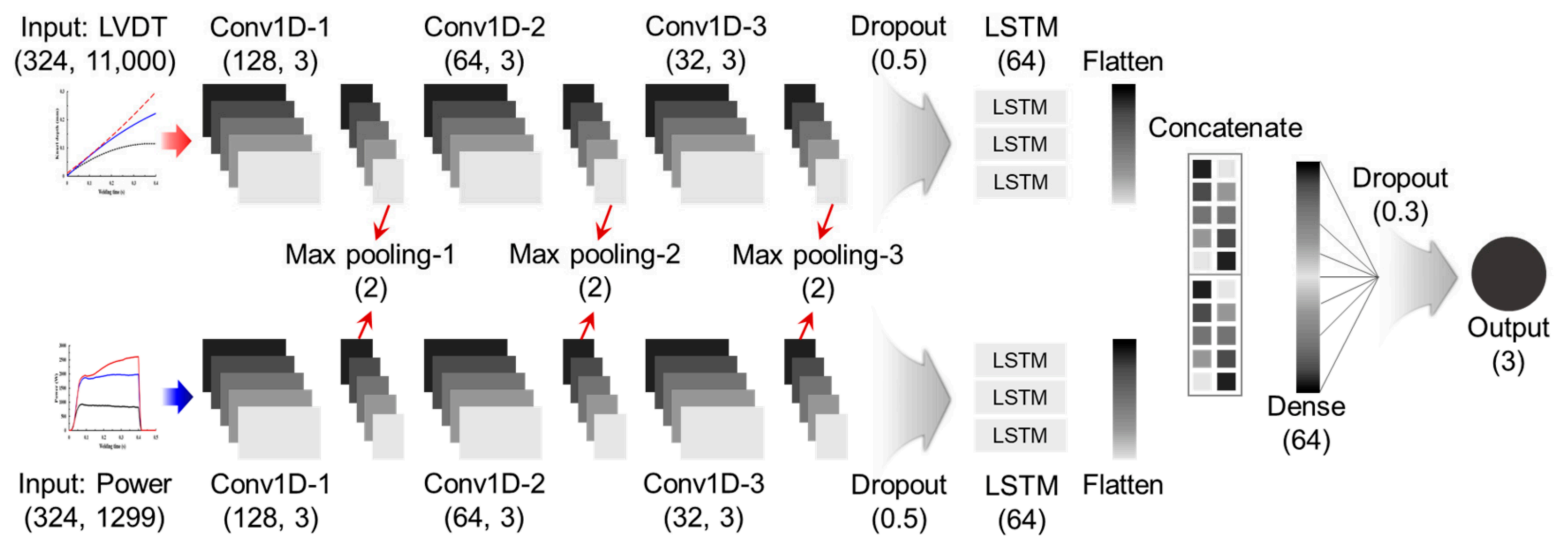
Architecture of CNN-LSTM model.

Figure 11 shows the structure of the 1D-CNN model; the time-series data for LVDT (324 × 11,000) and Power (324 × 1299) signals as input data are used to extract local features through a 1D-CNN layer. The applied model has a parallel structure that learns different input data independently to minimize bias for specific data. The CNN layer for the LVDT and power signals consists of three layers, with filter sizes of 128, 64, and 32, while the kernel size remains the same at 3 for each layer. The max-pooling layer was used to reduce the size of the feature map in order to decrease computational complexity and preserve important information. Subsequently, a dropout layer was used to prevent the model from overfitting to the training data and to improve its generalization performance. Additionally, in the process of learning long sequence data, there are limitations in capturing long-term dependencies due to vanishing and exploding gradients. Therefore, an LSTM layer was applied to delete unnecessary data and retain only important information; this learning model allows for the effective learning of changes and patterns over time. After undergoing the flattening process, the multi-dimensional feature map is transformed into a one-dimensional format, and the features extracted from the LVDT and power signals are combined into a single tensor through concatenation. The final dense layer used the ReLU activation function, and the output layer used the softmax function to develop the classification model. The optimization function used the Adam optimizer with learning rate, Beta_1, Beta_2, and Epsilon set to 0.001, 0.9, 0.999, and 1 × 10^−8^, respectively. Finally, the data split for training, validation, and testing was applied as 60%, 20%, and 20% to develop the classification model.

All Conv1D layers were implemented with a stride of 1 and valid padding. The LSTM layers consisted of 64 units with return_sequences = True, retaining the full temporal sequence before the Flatten operation. Detailed output shapes and trainable parameters for each layer are summarized in Table 6. The dataset was randomly divided using a fixed random seed (random_state = 42) without stratification, and no class weighting was applied during model training.

**Figure 12:**
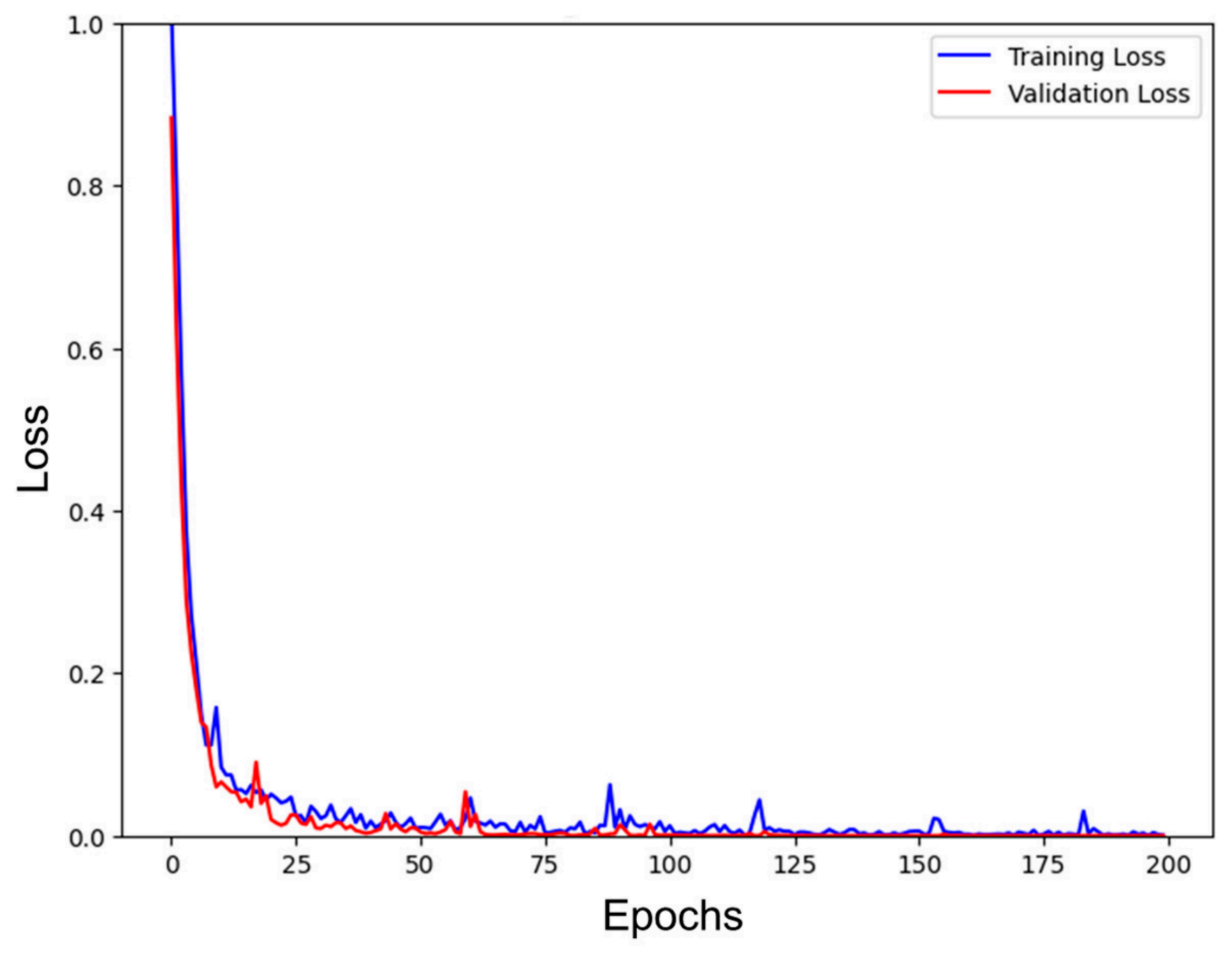
Training and validation loss curve of CNN-LSTM model.

Figure 12 shows the training and validation loss curves over 200 epochs. Both losses decrease rapidly during the initial epochs as the model learns underlying patterns, and subsequently stabilize at very low levels. The close alignment between the training and validation curves, with no significant divergence or instability, indicates that the model generalizes well without severe overfitting. Overall, the steady progression of the loss curves confirms successful and reliable model training.

### 3.5. Classification Performance and Model Evaluation

The generalization accuracy and predictive reliability of the proposed dual-branch 1D CNN–LSTM framework were quantitatively evaluated on the independent test dataset (65 samples) using confusion matrix analysis alongside Precision, Recall, Accuracy, and F1-score metrics. The classification performance metrics are mathematically defined as follows:

Precision = TP/(TP + FP)(2)

Recall = TP/(TP + FN)(3)

Accuracy = (TP + TN)/(TP + TN + FP + FN)(4)

F1-score = 2 · (Precision · Recall)/(Precision + Recall),(5)

where TP, TN, FP, and FN represent true positives, true negatives, false positives, and false negatives, respectively.

**Figure 13:**
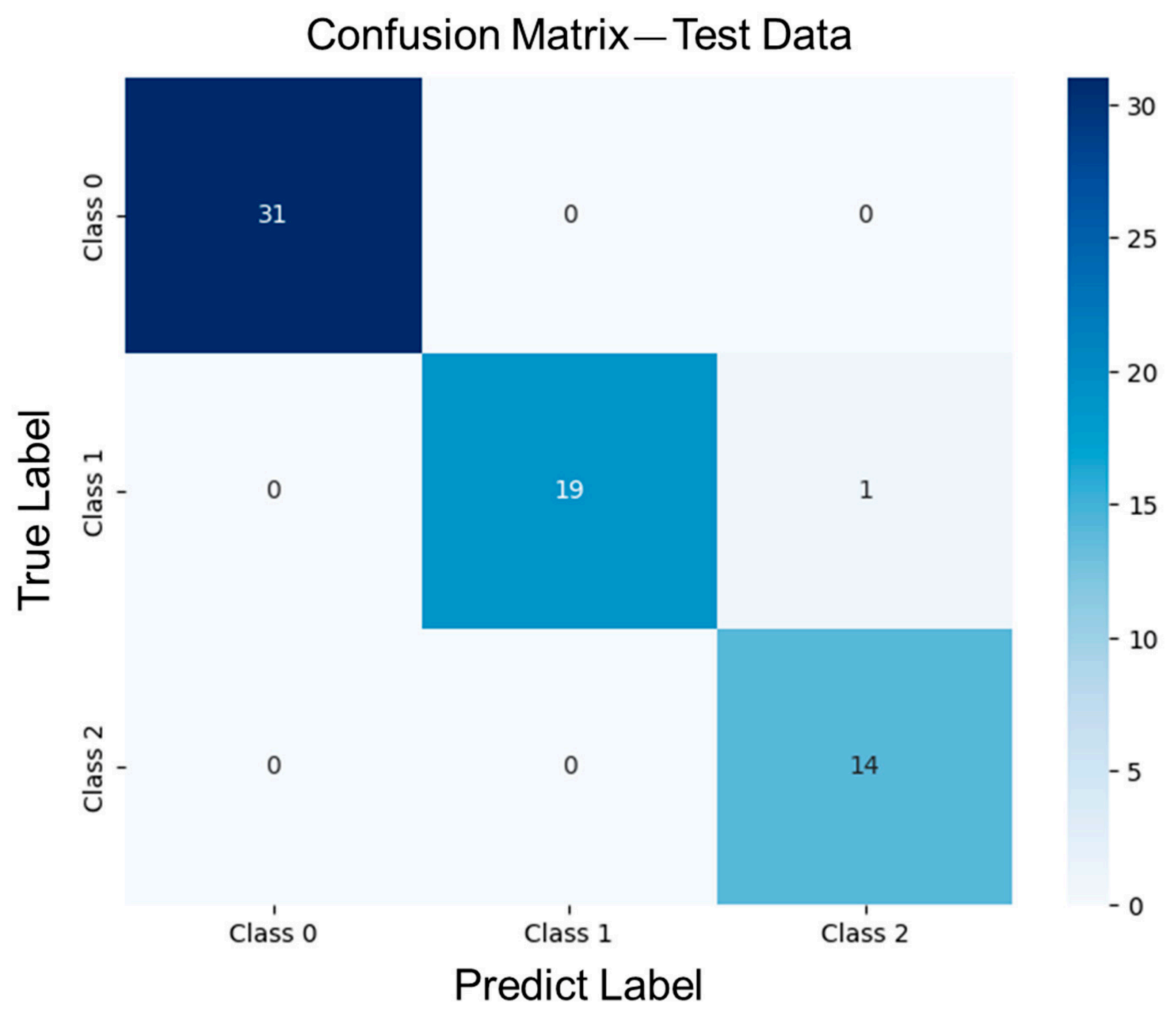
Confusion matrix of CNN-LSTM model.

The resulting confusion matrix and comprehensive performance statistics are presented in Figure 13 and Table 7. The proposed model correctly predicted 64 out of 65 unseen test instances, achieving an overall classification accuracy of 98.46% (~98%) with a macro-averaged F1-score of 0.98. Specifically, the model achieved perfect recognition for under-welding (Class 0, Precision: 1.00, Recall: 1.00) and over-welding (Class 2, Recall: 1.00). Only a single boundary sample from the normal welding class (Class 1) was misclassified into the over-welding regime, demonstrating remarkable performance across multi-layered foil joining states.

## 4. Conclusions

This study demonstrated a 1D CNN–LSTM framework system for quality classification in ultrasonic metal welding (UMW) of 60-layer pure Cu foil stacks (8 µm each). The conclusions are summarized as follows:

1. A synchronized sensing system was implemented using welder power signals and an LVDT displacement sensor mounted on the sonotrode housing. The measured vertical displacement exhibited a strong linear correlation with actual knurl penetration and weld indentation depths, validating its effectiveness as a direct indicator of dynamic foil stack compaction.
2. Joint quality was categorized into three distinct regimes based on delivered welding energy: under-welding (<500 J), normal welding (500–900 J), and over-welding (>900 J). Each regime exhibited characteristic dynamic power profiles and distinct penetration depth ratios (<30%, 30–50%, and >50% of the total stack thickness).
3. The 1D CNN–LSTM model was developed to independently analyze LVDT and power time-series signals, effectively capturing both local transient patterns and long-term temporal dependencies. The model achieved an overall classification accuracy of 98.46% (macro F1-score: 0.98) and a 100% defect recall on the held-out test set.

## Figures and Tables

**Table 1:** Specifications of the ultrasonic generator data logging system.

Parameter	Value
Sampling rate	1 kS/s
Temporary buffer storage	Up to 5000 datasets
Parallel processing capacity	Up to 16 generators

**Table 2:** Specifications of the LVDT displacement sensor and digital indicator module.

Subsystem/Unit	Parameter	Specification/Value
LVDT Probe Sensor (DP-10)	Measurement stroke range	0 to 10.45 mm
	Repeatability	0.5 μm
	Nonlinearity	0.1% (at ±1.0 mm)
	Measuring force	30 to 80 g
	Probe external diameter	Ø 8.0 mm
	Sensor mass	~20 g
Digital Indicator (DI-20W)	Excitation voltage	AC 0 to 2 Vrms
	A/D converter resolution	24-bit, 200 sps
	D/A converter resolution	16-bit, 200 Hz
	Analog output range	DC 0 to 10 V/4 to 20 mA
	Power supply	AC 90 to 240 V

**Table 3:** Technical specifications of the data acquisition (DAQ) module (NI USB-6001).

Parameter Category	Attribute/Feature	Specification
Analog Input	ADC resolution	14-bit
	Maximum sampling rate	20 kS/s
	Input voltage range	±10 V
	Absolute accuracy	6.0 mV
	System RMS noise	0.7 mV
	Small-signal bandwidth	300 kHz
Analog Output	DAC resolution	14-bit
	Update rate	5 kS/s
	Output range/Current	±10 V/±5 mA
Timing and Clock	Base clock frequency	80 MHz
	Frequency accuracy	±100 ppm
	Time resolution	12.5 ns

**Table 4:** Energy-based joint quality classification using the machine-reported welding energy supported by the corresponding indentation morphology and cross-sectional integrity [7].

Classification	Insufficient Welding Energy	Adequate Welding Energy	Excessive Welding Energy
Welding Energy (J)	Below 500	500~900	Above 900
**Appearance**	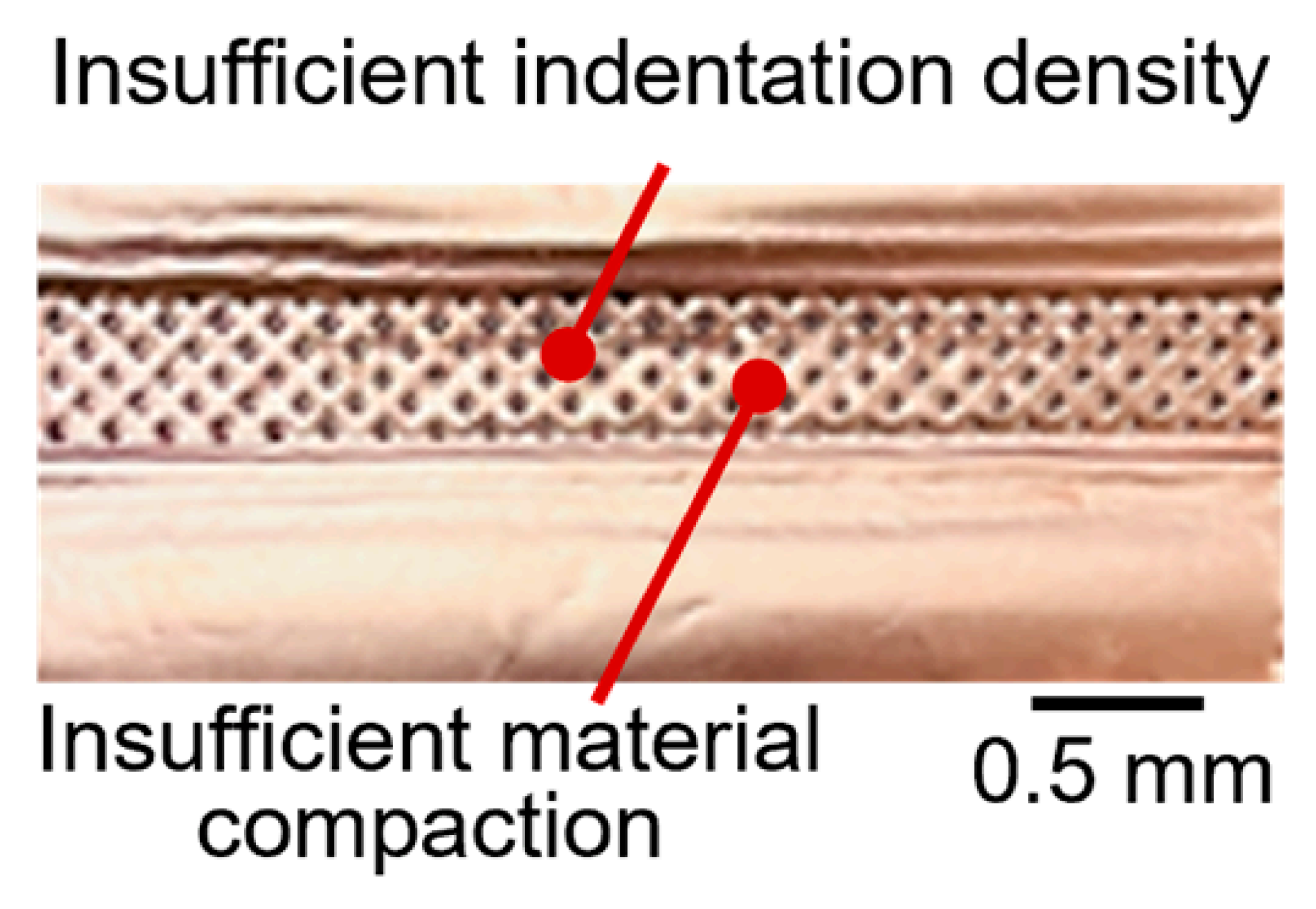	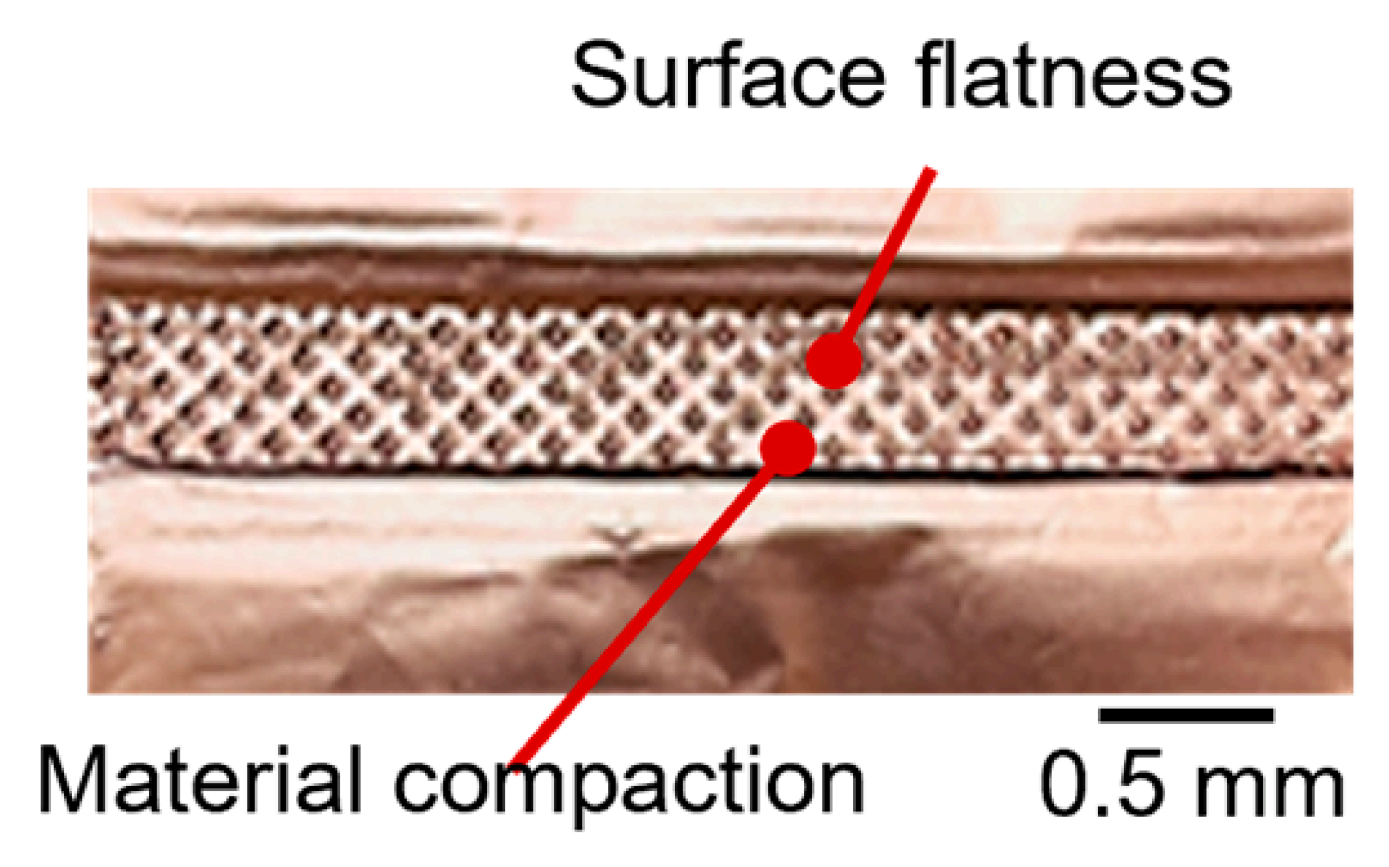	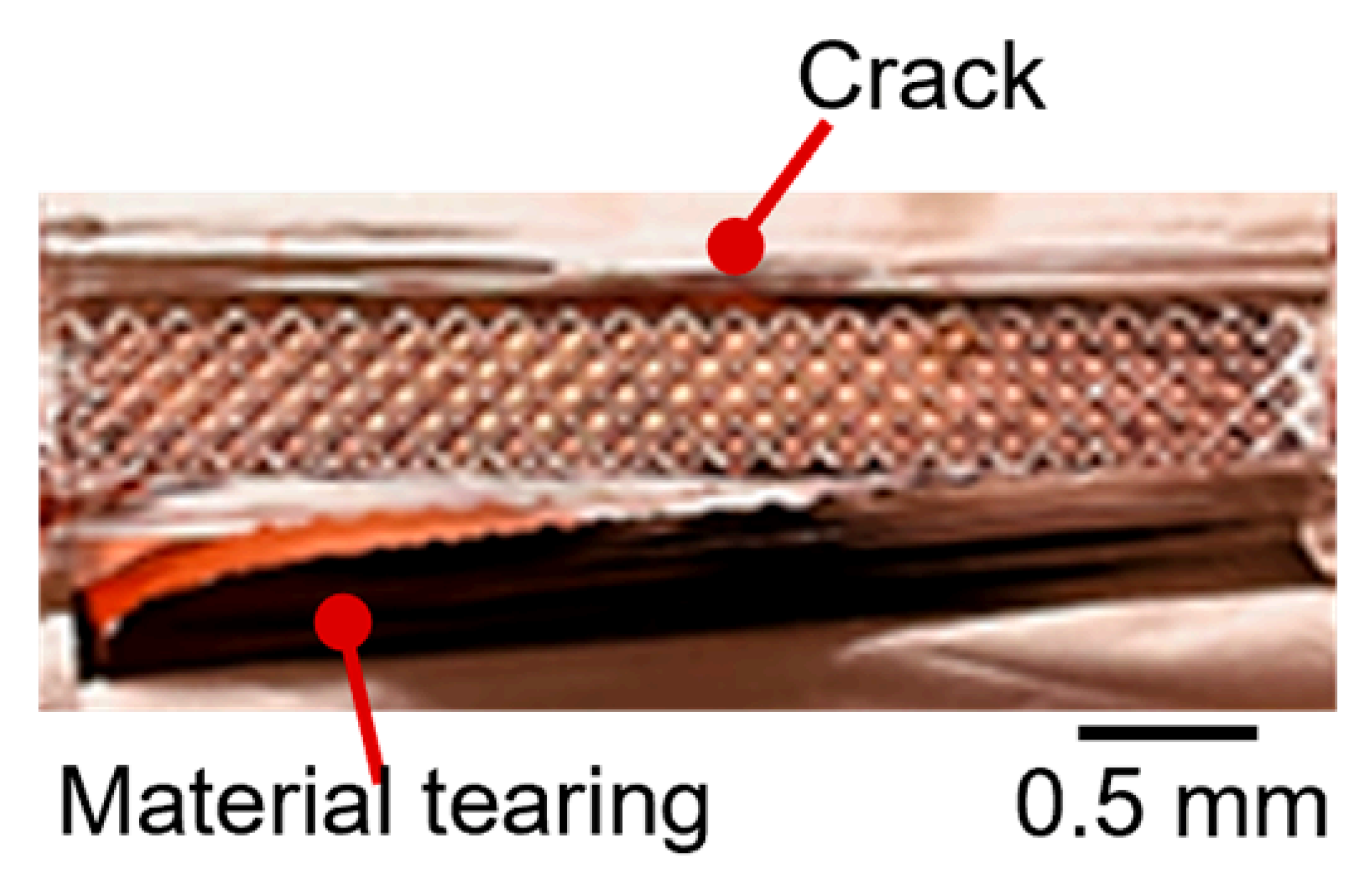
**Indentation length (mm)**	0.73~1.10 (D_1_)	1.10~1.28 (D_1_)	1.28~1.79 (D_1_)
**Indentation depth (mm)**	0.05~0.18 (Avg.)	0.18~0.22 (Avg.)	0.22~0.53 (Avg.)

**Table 5:** Classification of multi-layered Cu joint quality based on delivered ultrasonic welding energy.

Quality Class	Label	Clamping Pressure (Bar)	Vibrational Amplitude (μm)	Welding Time (s)	Welding Energy Range (J)
Under-welding	Class 0	0.5	15, 20	0.2, 0.4, 0.6	E < 500
		0.5	25	0.2, 0.4	
		1.0	15	0.2, 0.4, 0.6	
		1.0	20	0.2, 0.4	
		1.0	25	0.2	
		1.5	15	0.2, 0.4	
		1.5	20, 25	0.2	
Normal welding	Class 1	0.5	20	0.8	500 ≤ E ≤ 900
		0.5	25	0.6	
		1.0	15	0.8	
		1.0	20	0.6	
		1.0	25	0.4	
		1.5	15	0.6, 0.8	
		1.5	20, 25	0.4	
Over-welding	Class 2	0.5	25	0.8	E > 900
		1.0	20	0.8	
		1.0	25	0.6, 0.8	
		1.5	20, 25	0.6, 0.8	

**Table 6:** Detailed architecture and parameters of the proposed two-branch 1D CNN-LSTN model.

Branch	Layer	Output Shape	Parameters
LVDT	Input	(5879, 1)	0
	Conv1D, 128, k = 3	(5877, 128)	512
	MaxPooling1D, pool = 2	(2938, 128)	0
	Conv1D, 64, k = 3	(2936, 64)	24,640
	MaxPooling1D, pool = 2	(1468, 64)	0
	Conv1D, 32, k = 3	(1466, 32)	6176
	MaxPooling1D, pool = 2	(733, 32)	0
	Dropout (0.2)	(733, 32)	0
	LSTM, 64	(733, 64)	24,832
	Flatten	(46,912)	0
Power	Input	(1299, 1)	0
	Conv1D, 128, k = 3	(1297, 128)	512
	MaxPooling1D, pool = 2	(648, 128)	0
	Conv1D, 64, k = 3	(646, 64)	24,640
	MaxPooling1D, pool = 2	(323, 64)	0
	Conv1D, 32, k = 3	(321, 32)	6176
	MaxPooling1D, pool = 2	(160, 32)	0
	Dropout (0.2)	(160, 32)	0
	LSTM, 64	(160, 64)	24,832
	Flatten	(10,240)	0
Fusion	Concatenate	(57,152)	0
	Dense, 64	(64)	3,657,792
	Dropout (0.3)	(64)	0
Output	Dense, Softmax	(3)	195

**Table 7:** Performance metrics of CNN-LSTM model.

Metrics	Precision	Recall	F1-Score	Support
Class 0	1.00	1.00	1.00	31
Class 1	1.00	0.95	0.97	20
Class 2	0.93	1.00	0.97	14
Accuracy	-	-	0.98	65
Macro avg.	0.98	0.98	0.98	65
Weight avg.	0.98	0.98	0.98	65

## Data Availability

The original contributions presented in this study are included in the article. Further inquiries can be directed to the corresponding author.
